# Fecal carriage of multidrug-resistant Enterobacterales and its associated factors among hospital and non-hospital janitors at the University of Gondar, Northwest Ethiopia: a comparative cross-sectional study

**DOI:** 10.1038/s41598-026-55339-6

**Published:** 2026-06-04

**Authors:** Amanuale Zayede, Yitayih Wondimeneh, Sirak Biset, Eshet Gebrie, Mucheye Gizachew

**Affiliations:** 1 Department of Medical Microbiology, School of Biomedical and Laboratory Sciences, College of Medicine and Health Sciences, University of Gondar, Gondar, Ethiopia; 2 Department of Medical Laboratory Sciences, College of Health Sciences, Woldia University, Woldia, Ethiopia; 3 Department of Clinical Chemistry, School of Biomedical and Laboratory Sciences, College of Medicine and Health Sciences, University of Gondar, Gondar, Ethiopia

**Keywords:** Fecal carriage, Enterobacterales, Enterobacteriaceae, AMR, MDR, Hospital janitors, Non-hospital janitors, UoG, Diseases, Health care, Medical research, Microbiology

## Abstract

Multidrug-resistant (MDR) Enterobacterales represent a severe global health challenge. Janitors, who are frequently in contact with contaminated surfaces, may become carriers and spreaders of these pathogens. This risk is especially acute in resource-constrained environments such as Ethiopia, where research into this potential transmission route remains scarce. The aim of this study was to assess fecal carriage rates of multidrug-resistant Enterobacterales and its associated factors among hospital and non-hospital janitors at the University of Gondar (UoG), Northwest Ethiopia. A comparative cross-sectional study was conducted from June 15 to September 25, 2025 at the UoG. A total of 385 janitors (203 hospital and 182 non-hospital) were selected using a stratified and simple random sampling techniques. Data on socio-demographic and associated factor were collected using a semi-structured questionnaire. Stool samples were collected aseptically, cultured on MacConkey agar, and identified using biochemical test results. Antimicrobial resistance (AMR) patterns were determined by the Kirby-Bauer disk diffusion method following Clinical and Laboratory Standards Institute (CLSI) 2024 guidelines. Quality control was ensured by using standard reference strain (*E. coli* ATCC 25922). Data analysis was performed using SPSS version 27.0, employing chi-square tests and logistic regression. A *p*-value of < 0.05 was considered statistically significant. Among the 385 stool samples, a total of 416 Enterobacterales isolates were recovered, 229 from hospital janitors and 187 from non-hospital janitors. The overall fecal carriage rate of MDR Enterobacterales among janitors was 54.0% (95% CI 48.9, 59.1). This rate was significantly higher in hospital janitors compared to non-hospital janitors ((63.5% (95% CI 56.5, 70.2) vs. 43.4% (95% CI 36.1, 50.9), respectively; *p* < 0.001)). History of antibiotic use, urinary tract infection, and gastrointestinal tract symptom in the last three months were significantly associated with MDR Enterobacterales carriage among janitors. The high carriage rates of MDR Enterobacterales identified among janitors suggest that janitors may serve as a potential reservoir for resistant bacteria. To address this, we recommend their inclusion in national antimicrobial resistance surveillance programs and the implementation of tailored infection prevention and control training.

**Supplementary Information:**

The online version contains supplementary material available at 10.1038/s41598-026-55339-6.

## Introduction

Enterobacterales, a large, diverse order of Gram-negative bacteria, includes pathogens like *Escherichia*, *Klebsiella*, and *Shigella*. They cause diverse infections (e.g., urinary, respiratory, bloodstream) in both community and healthcare settings^1,2^.

The rise of antimicrobial resistance (AMR) among these bacteria has emerged as a critical global challenge, with many now classified as multidrug-resistant (MDR). This resistance complicates standard treatments and is associated with severe outcomes, including increased morbidity, mortality, prolonged hospitalization, and greater healthcare costs^3–6^. Notably, the Infectious Diseases Society of America (IDSA) has identified *E. coli* and *K. pneumoniae* as priority pathogens for which new antibiotics are urgently needed^7^.

Multidrug-resistant (MDR) Enterobacterales are disseminated via numerous reservoirs, including colonized individuals, contaminated medical instruments, hospital wastewater systems, drains, sewage, and food sources^8,9^. The intestinal microbiome also harbors resistance determinants, enabling their horizontal transfer to pathogenic species^10^.

Among occupational groups at risk, janitors are routinely exposed to a wide array of contaminants, cleaning agents, moisture, dust, and polluted air, leading to elevated risks of respiratory and dermatological conditions^11^. Despite their essential role in infection control, janitors often occupy low-wage, low-status positions and face multiple physical, chemical, biological, and psychosocial hazards^12,13^.

A study involving janitors in Saudi Arabia found that the colonization rate of Enterobacteriaceae was 15.6%, with *Klebsiella* species being the most commonly isolated. Those working in high-risk hospital areas, such as intensive care units (ICUs) and operating rooms, are especially vulnerable to colonization by Enterobacteriaceae^14^. Similarly, a cross-sectional study of 456 multinational migrant food handlers in Qatar (2015–2016) found that 17.1% were positive for *E. coli*, of which 27% exhibited MDR^15^.

Janitors are at high risk of exposure to contaminants such as cleaning agents, polluted air, dust, and through wet work. Furthermore, janitors working in hospitals may acquire MDR organisms from the clinical environment and potentially disseminate them into the community. Reports In Ethiopia showed that, gastrointestinal colonization by antimicrobial-resistant Enterobacterales has risen markedly in recent years^16^. However, in the study setting, there is a scarcity of data directly comparing the fecal carriage of MDR Enterobacterales among janitors in hospital versus non-hospital settings. Therefore, these findings address a critical knowledge gap by identifying janitors as a potential reservoir for MDR Enterobacterales, and this data also a vital for researchers designing targeted surveillance and future interventions.

This study therefore aims to assessed the fecal carriage of MDR Enterobacterales, and to identify associated factors among janitors working in both hospital and non-hospital settings at the University of Gondar (UoG).

## Materials and methods

### Study design and period, and setting

From June to September 2025, a comparative cross-sectional study was carried out among hospital and non-hospital janitors at the UoG in Northwest Ethiopia. UoG, a most prominent higher education institution situated in Gondar city, hosts five campuses, including the College of Medicine and Health Sciences (CMHS)), Maraki, Atse Tewodros, Atse Fasile, and Tseda^17^. The CMHS includes the University of Gondar Comprehensive Specialized Hospital (UoGCSH), which serves as a 960-bed referral facility for over 13 million people. Annually the hospital manages approximately 400,000 outpatient visits and 30,000 admissions. It has , 28 wards, 15 outpatient departments, and a central bacteriology laboratory within the Department of Medical Microbiology^18^. Currently, UoG employs 710 janitors, 400 of whom work within the hospital area.

### Population

#### Source population

All hospital and non-hospital janitors working at the UoG were the source population.

#### Study population

All hospital and non-hospital janitors working at the UoG during the data collection period and who met the inclusion criteria were the study population.

### Eligibility criteria

#### Inclusion criteria

**Hospital janitors:** who have been employed at the UoGCSH for at least three months prior to data collection were included.

**Non-hospital janitors:** who have been employed in non-hospital working areas of the UoG for at least three months prior to data collection were included.

#### Exclusion criteria

**Hospital janitors:** Janitors employed by the UoGCSH and are working in its administration building, cafeteria and gardens were excluded. In addition, janitors who have taken antibiotics within the past one week before the start of data collection were excluded.

**Non-hospital janitors:** Janitors employed by the UoG and have a history of working in hospital settings within the last six months prior to data collection were excluded^19,20^. In addition, janitors who have taken antibiotics within the past one week before the start of data collection were excluded.

### Variables

#### Dependent variables

- Antimicrobial resistance patterns of Enterobacterales isolates, and
- Fecal carriage of MDR Enterobacterales were dependent variables.

#### Independent variables

##### Sociodemographic variables

Age, sex, educational status, marital status, family size, living with children.

##### Occupation related factors

UoG working area, department, work shift, service years, training regarding to IPC, access to appropriate PPE, access to handwashing facilities.

##### Hygiene related factors

Use gloves while cleaning, exposure to contaminated bodily fluids during cleaning procedures, hand washing habit at work place, methods used for hand cleaning, access to clean and safe toilet facilities at home, practice hand hygiene at home, fingernail status, source of water for drinking.

##### Behavior related factors

Use of antibiotics without prescription, drinking unpasteurized milk, eating raw meat, eating raw vegetables.

##### Clinical related factors

History of antibiotics use, hospital admission, urinary tract infection, diarrhea, close contact with a hospitalized person in the last three months, and having chronic disease were independent variables^21–23^.

### Sample size and sampling technique

#### Sample size determination

The sample size was calculated using the formula for comparing two independent proportions^24^. A prevalence rate of 50% was assumed for both groups, as no previous studies have been conducted in Ethiopia regarding to fecal carriage of MDR Enterobacterales among hospital and non-hospital janitors.

The minimum sample size is calculated as follows.

$${\text{n }} = \left( {{\mathrm{Z}}\alpha /_{{2}} + {\text{ Z}}_{\beta } } \right)^{{2}} * \, \left( {{\mathrm{P}}_{{1}} \left( {{1} - {\text{ P}}_{{1}} } \right) \, + {\text{ P}}_{{2}} \left( {{1} - {\text{ P}}_{{2}} } \right)} \right)$$

$$\left( {{\mathrm{P}}_{{1}} - {\text{ P}}_{{2}} } \right)^{{2}}$$

Were;

n = required sample size for each group.

α = Type I error (level of significant).

β = Type Ⅱ error (1- β = power of the study).

Zα/_2_ = Z-value corresponding to the confidence level (for 95% confidence, Zα/_2_ = 1.96).

Z_β_ = Z-value corresponding to the power (for 80% power, and Z_β_ = 0.84).

Since, there is no previous data, based on assumption set P₁—P₂ = 10% (a small detectable difference). P₁ = 50%, P₂ = 40% (10% difference)

$${\text{n }} = \left( {{1}.{96 } + \, 0.{84}} \right)^{{2}} * \, \left( {0.{5 }\left( {{1} - \, 0.{5}} \right) \, + \, 0.{4 }\left( {{1} - \, 0.{4}} \right)} \right) \approx {\text{385 per group}}$$

$$\left( {0.{1}} \right)^{{2}}$$

A finite population correction was applied separately for each group due to the small total populations (hospital janitors, N = 400; non-hospital janitors, N = 310). The corrected sample size for each group was calculated using the formula: n *adjusted* = n / [1 + (n—1)/N].

For hospital janitors: n *adjusted* = 385 **/** [1 + (385- 1)/400] ≈197.

For non-hospital janitors: n *adjusted* = 385 **/** [1 + (385- 1)/310] ≈172.

Considering, a 10% non-response rate (n adjusted = n initial /1 − non-response rate), the final minimum sample size is set at 411, with 219 hospital and 192 non-hospital janitors.

#### Sampling technique

**Fig. 1:**
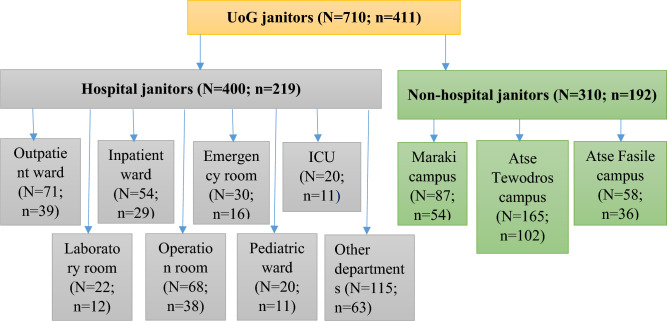
Flow of study participant selection using stratified and simple random sampling technique.

Study participants were selected using stratified and simple random sampling techniques (Fig. 1).

A full list of janitors was obtained from the UoG Human Resource Directorate and stratified into hospital and non-hospital groups. The sample size was proportionally distributed across the two strata based on their respective populations. Within each stratum, participants were selected using a simple random sampling technique via the lottery method: names or identification numbers were written on slips of paper, placed in a container, and drawn randomly until the required sample size per group was met.

#### Data collection tool and procedure

Socio-demographic, hygiene, behavioral, occupational, and clinical data related to MDR Enterobacterales colonization were gathered via face-to-face interviews using a semi-structured questionnaire (Supplementary Text S1).

Participants were then asked to provide approximately 2 g of fresh stool. They placed clean newspaper over the toilet seat, used a wooden stick to transfer a pea-sized portion, especially from any bloody, slimy, or watery areas into a sealed, leak-proof container. Urine contamination was avoided by voiding beforehand, and handwashing with soap and water was required before and after collection.

If culture was not possible within two hours, a sterile swab was inserted into the sample, rotated, and placed into Cary-Blair medium (HI MEDIA Laboratories, Mumbai, India). Each container was labeled with the sample number, date, and time, kept in a cold box, and transported to the Medical Microbiology Laboratory at the School of Biomedical and Laboratory Sciences (SBLS) for analysis^25,26^. Two trained laboratory technologists collected all data.

### Laboratory methods

#### Enterobacterales isolation and identification

Stool samples collected from each participant were inoculated onto MacConkey agar (Oxoid Ltd, Basingstoke, UK), which were incubated aerobically at 37 °C for 18 to 24 h. Following incubation, all plates were examined for bacterial growth. Subsequently, sub-culturing on fresh MacConkey agar (Oxoid Ltd, Basingstoke, UK) was performed as necessary to obtain isolated, pure colonies for further characterization^27^. The isolates were then identified based on a combination of Gram staining (showing Gram-negative rods), colony morphology, and biochemical test results^28^. A series of biochemical tests (all from Oxoid Ltd, Basingstoke, UK) were included: Triple Sugar Iron agar to assess glucose/lactose/sucrose fermentation, gas production, and H₂S production, lysine decarboxylase activity, indole production, citrate utilization, urea hydrolysis, and motility test (Supplementary Text S2)^28^.

#### Antimicrobial susceptibility test of isolates

Antimicrobial susceptibility test (AST) was performed using the Kirby-Bauer disk diffusion method. The inoculum suspension was prepared and compared to a 0.5 McFarland turbidity standard. Mueller Hinton agar (MHA, Oxoid Ltd, Basingstoke, UK) plates were inoculated using the lawn culture method. The following antimicrobial disks (all from Oxoid Ltd, Basingstoke, UK) were applied to the MHA plates, which were incubated aerobically at 35 °C ± 2 °C for 16 to 18 h: Amoxicillin-Clavulanic acid (20/10 µg), cefotaxime (30 μg), ceftazidime (30 μg), ceftriaxone (30 μg), cefoxitin (30 µg), meropenem (10 μg), imipenem (10 μg), gentamicin (10 μg), Amikacin (30 μg), Tetracycline (30 μg), Chloramphenicol (30 μg), Ciprofloxacin (5 μg), and trimethoprim-sulfamethoxazole (1.25/23.75 μg)^29,30^. The results were interpreted as susceptible, intermediate, or resistant according to the Clinical and Laboratory Standards Institute (CLSI) 2024 guidelines (Supplementary Table S3)^29^. The MDR patterns of the isolates were identified using the criteria established by Magiorakos et al.^31^.

#### Data quality measures

The questionnaire was originally prepared in English, and then translated into local language (Amharic) for data collection, which was then translated back into English by an independent translator for analysis and reporting. A pre-test was conducted on 5% of the calculated sample size among janitors at the UoG, CMHS campus to assess validity and clarity. Close supervision was maintained by the principal investigator (PI) throughout the data collection period.

All laboratory procedures of sample collection, handling, storage, and transport were performed according to standard operating procedures (SOPs) and manufacturer recommendations. The sterility of newly prepared culture media was assessed by randomly selecting 5% of each batch, incubating them at 35 °C ± 2 °C for 16 to 18 h, and confirming the absence of microbial growth. The performance of newly prepared culture media was verified using the *E. coli* American Type Culture Collection (ATCC) 25922 standard strain. Additionally, the quality of antimicrobial disks was checked using the *E. coli* ATCC 25922 (Supplementary Table S4). Furthermore, the viability of the 15% glycerol-TSB medium was tested by confirming the growth of *E. coli* ATCC 25922 both before and after storage. All confirmed Enterobacterales isolates were stored at −20 °C in 15% glycerol-TSB^25^.

#### Data management and analysis

All data were entered and managed using EpiData EntryClient and Manager, version 4.6.0.2 (EpiData Association, Odense, Denmark). Data were then exported to Statistical Package for the Social Sciences (SPSS), version 27.0 (IBM Corp., Armonk, NY, USA) for statistical analysis.

Descriptive statistics (frequencies and percentages) were computed to summarize the characteristics of the study population. Chi-square tests were used to assess for statistically significant differences in Enterobacterales isolates profiles, and AMR between the hospital and non-hospital janitors. The association between independent variables and the dependent variables (MDR Enterobacterales) was analyzed using bivariable and multivariable logistic regression models. Variables with a *p*-value of ≤ 0.25 in the bivariable analysis were included in the multivariable analysis. Prior to running the multivariable logistic regression model, multicollinearity among independent variables was checked using variance inflation factor (VIF) and tolerance values, and model fitness was assessed using the Hosmer–Lemeshow goodness-of-fit test. The results of these associations are presented as Crude Odds Ratios (COR) and Adjusted Odds Ratios (AOR) with corresponding 95% confidence intervals (CI). A *p*-value of < 0.05 was considered statistically significant.

#### Operational definitions

##### Multidrug-resistant (MDR)

A bacteria that have developed non-susceptibility to at least one agent in three or more antimicrobial categories^31^.

##### Hospital janitors

A personnel who are responsible for maintaining hygiene and preventing infections in healthcare facilities, they might work across an entire hospital or specialize in one specific department, may work full-time or part-time. This group includes janitors who clean and disinfect patient rooms, operating theaters, corridors, and frequently touched surfaces^32^.

##### Non-hospital janitors

Also known as building service workers or custodians, are personnel responsible for maintaining cleanliness and hygiene in various settings outside healthcare facilities, such as administrative offices, schools, and public spaces to ensure a safe and sanitary environmen^33^.

### Ethical consideration

We conducted the study following the "Declaration of Helsinki"^34^. Ethical approval for the study was obtained from the Ethical Review Committee of the SBLS at the UoG, with reference number SBLS/997, date 05/06/2025 prior to the start of any research activities.

Participation in the study was voluntary. Written informed consent was obtained from each participant after a thorough explanation of the study’s purpose, procedures, risks, and benefits. For participants who were unable to read, the information sheet was read to them in the presence of a witness, and they provided a fingerprint or a mark, which was countersigned by the witness. No minors were involved in this study.

Janitors who were found to be positive for fecal carriage of target bacteria were referred to their respective staff medical centers for further diagnosis and appropriate health education on IPC. The confidentiality of all participants was maintained throughout the study. All laboratory test results were used solely for the purposes of this research. Identifying information, such as names, was not collected on the data collection forms (Supplementary Text S1).

## Results

### Sociodemographic characteristics of study participants

The initial calculated sample size was 411. With a total response rate of 93.67%, the study was finally conducted among 385 participants (203 hospital janitors and 182 non-hospital janitors). The predominant study participants were females 373 (96.88%), aged between 20 and 51 years. The mean age was higher among hospital janitors (31.5 ± 5.99 years), compared to non-hospital janitors (29.13 ± 5.45 years). Over half of the participants, 215 (55.84%), held a diploma or higher degree, and 216 (56.1%) had more than five years of work experience. The majority were married 239 (62.08%), lived with their children 236 (61.30%), and had a family size of 1–5 people 368 (95.58%) (Table 1).

**Table 1:** Sociodemographic characteristics of hospital (N = 203) and non-hospital janitors (N = 182) at the UoG, Northwest Ethiopia, June to September 2025.

Variables	Categories	Study Participants		Total (N = 385); n (%)
		Hospital Janitors (N = 203); n (%)	Non-hospital Janitors (N = 182); n (%)	
Gender	Male	10 (4.93)	2 (1.10)	12 (3.12)
	Female	193 (95.07)	180 (98.90)	373 (96.88)
Age	18–28	75 (36.90)	117 (64.30)	192 (49.9)
	29–39	113 (55.70)	56 (30.80)	169 (43.9)
	≥ 40	15 (7.40)	9 (4.90)	24 (6.2)
Level of education	No formal education	3 (1.48)	4 (2.20)	7 (1.82)
	Primary school	13 (6.4)	8 (4.40)	21 (5.45)
	Secondary school	73 (35.96)	69 (37.91)	142 (36.88)
	Diploma and above	114 (56.16)	101 (55.49)	215 (55.84)
Marital status	Single	57 (28.08)	58 (31.87)	115 (29.87)
	Married	122 (60.10)	117 (64.29)	239 (62.08)
	Divorced	18 (8.87)	7 (3.85)	25 (6.49)
	Widowed	6 (2.96)	0	6 (1.56)
Family size	1–5	188 (92.6)	180 (98.9)	368 (95.58)
	> 5	15 (7.40)	2 (1.1)	17 (4.42)
Living with children’s	Yes	134 (66.01)	102 (56.04)	236 (61.30)
	No	69 (33.99)	80 (43.96)	149 (38.70)
Service years	1–5	79 (38.9)	90 (49.5)	169 (43.91)
	> 5	124 (61.1)	92 (50.5)	216 (56.1)

### Occupational and hygiene related characteristics of study participants

A higher proportion of hospital janitors reported receiving IPC training, 124 (61.08%) compared to 28 (15.38%), non-hospital janitors. Additionally, a greater percentage of hospital janitors had non-trimmed fingernails, 66 (32.51%) compared to 33 (18.13%), non-hospital janitors (Table 2).

**Table 2:** Occupational and hygiene related characteristics of hospital (N = 203) and non-hospital janitors (N = 182) at the UoG, Northwest Ethiopia, June to September 2025.

Variables	Categories	Study participants		Total (N = 385); n (%)
		Hospital janitors (N = 203); n (%)	Non-hospital janitors (N = 182); n (%)	
Receiving IPC training	Yes	124 (61.08)	28 (15.38)	152 (39.48)
	No	79 (38.92)	154 (84.62)	233 (60.52)
Access to PPE	Yes	157 (77.34)	79 (43.41)	236 (61.30)
	Sometimes	33 (16.26)	101 (55.49)	134 (34.81)
	No	13 (6.40)	2 (1.10)	15 (3.90)
Hand washing access	Always available	150 (73.89)	76 (41.76)	226 (58.70)
	Often unavailable	18 (8.87)	101 (55.49)	119 (30.91)
	No access	35 (17.24)	5 (2.75)	40 (10.39)
Use glove during cleaning	Always	197 (97.04)	174 (95.60)	371 (96.36)
	Only for dirty tasks	4 (1.97)	6 (3.30)	10 (2.60)
	Rarely	2 (0.99)	2 (1.10)	4 (1.04)
	Never	0	0	0
Exposure to contaminated surfaces	Frequently	20 (9.85)	73 (40.11)	93 (24.16)
	Occasionally	137 (67.49)	40 (21.98)	177 (45.97)
	Never	46 (22.66)	69 (37.91)	115 (29.87)
Frequency of hand washing	Every hour	10 (4.93)	11 (6.04)	21 (5.45)
	After every cleaning task	189 (93.10)	171 (93.96)	360 (93.51)
	Only after used restroom and before eating	2 (0.99)	0	2 (0.52)
	Only when hands are visibly dirty	2 (0.99)	0	2 (0.52)
Hand cleaning soon after handling waste materials	Yes	163 (80.30)	170 (93.41)	333 (86.49)
	Sometimes	37 (18.23)	12 (6.59)	49 (12.73)
	No	3 (1.48)	0	3(0.78%)
Method of hand washing	Only with water	57 (28.08)	17 (9.34)	74 (19.22)
	With soap and water	146 (71.92)	163 (89.56)	309 (80.26)
	With alcohol based sanitizer	0	2 (1.10)	2 (0.52)
Fingernail status	Trimmed	137 (67.49)	149 (81.87)	286 (74.29)
	Non-trimmed	66 (32.51)	33 (18.13)	99 (25.71)
Safe toilet facility at home	Yes	174 (85.71)	167 (91.76)	341 (88.57)
	No	29 (14.29)	15 (8.24)	44 (11.43)
Hand hygiene practice at home	Yes	177 (87.19)	178 (97.80)	355 (92.21)
	Sometimes	19 (9.36)	4 (2.20)	23 (5.97)
	No	7 (3.45)	0	7 (1.82)
Source of water for drinking	Tap water	185 (91.13)	165 (90.66)	350 (90.91)
	Hand dug well water	18 (8.87)	17 (9.34)	35 (9.09)

*PPE* Personnel protective equipment, *IPC* Infection prevention and control.

### Behavioral and clinical characteristics of study participants

A higher proportion of hospital janitors reported use of prescribed antibiotics in the last three months, 100 (49.26%) compared to 50 (27.47%), non-hospital janitors. Furthermore, a greater percentage of hospital janitors had a history of GI symptom in the last three months, 74 (36.45%) compared to 26 (14.29%), non-hospital janitors. A similar disparity was observed for a history of UTI in the last three months, with hospital janitors 59 (29.06%), reporting a higher proportion than 23 (12.64%), non-hospital janitors (Table 3).

**Table 3:** Behavioral and clinical related characteristics of hospital (N = 203) and non-hospital janitors (N = 182) at the UoG, Northwest Ethiopia, June to September 2025.

Variables	Categories		Study participants		Total (N = 385); n (%)
			Hospital Janitors (N = 203); n (%)	Non-hospital janitors (N = 182); n (%)	
Antibiotics used without prescription in the last 3 months	Yes	< 1 week	34 (59.65)	37 (66.07)	71 (62.83)
		1–2 weeks	18 (31.58)	19 (33.93)	37 (32.74)
		> 2 weeks	5 (8.77)	0	5 (4.42)
		Total	57 (28.08)	56 (30.77)	113 (29.35)
	No		146 (71.92)	126 (69.23)	272 (70.65)
Drinking unpasteurized milk	Yes		60 (29.56)	44 (24.18)	104 (27.01)
	No		143 (70.44)	138 (75.82)	281 (72.99)
Eating raw meat	Yes		36 (17.73)	27 (14.84)	63 (16.36)
	No		167 (82.27)	155 (85.16)	322 (83.64)
Eating uncooked vegetables	Yes		76 (37.44)	43 (23.63)	119 (30.91)
	No		127 (62.56)	139 (76.37)	266 (69.09)
Used prescribed antibiotics in the last 3 months	Yes	Appropriately	30 (30.00)	27 (54.00)	57 (38.00)
		Not- appropriately	70 (70.00)	23 (46.00)	93 (62.00)
		Total	100 (49.26)	50 (27.47)	150 (38.96)
	No		103 (50.74)	132 (72.53)	235 (61.04)
History of GI symptom in the last 3 months	Yes		74 (36.45)	26 (14.29)	100 (25.97)
	No		129 (63.55)	156 (85.71)	285 (74.03)
History of UTI in the last 3 months	Yes		59 (29.06)	23 (12.64)	82 (21.29)
	No		144 (70.94)	159 (87.36)	303 (78.71)
History of chronic disease	Yes		14 (6.90)	17 (9.34)	31(8.05%)
	No		189 (93.10)	165 (90.66)	354 (91.95)
History of hospital admission in the last 3 months	Yes	Medical instrumentation	4 (11.40)	15 (71.40)	19 (33.90)
		No- Medical instrumentation	31 (88.60)	6 (28.60)	37 (66.10)
		Total	35 (17.24)	21 (11.54)	56 (14.55)
	No		168 (82.76)	161 (88.46)	329 (85.45)
Close contact with hospitalized person in the last 3 months	Yes		101 (49.75)	60 (32.97)	161 (41.82)
	No		102 (50.25)	122 (67.03)	224 (58.18)

*GI* gastrointestinal, *UTI* urinary tract infection.

### Fecal carriage rate of Enterobacterales isolates among hospital and non-hospital janitors

**Fig. 2:**
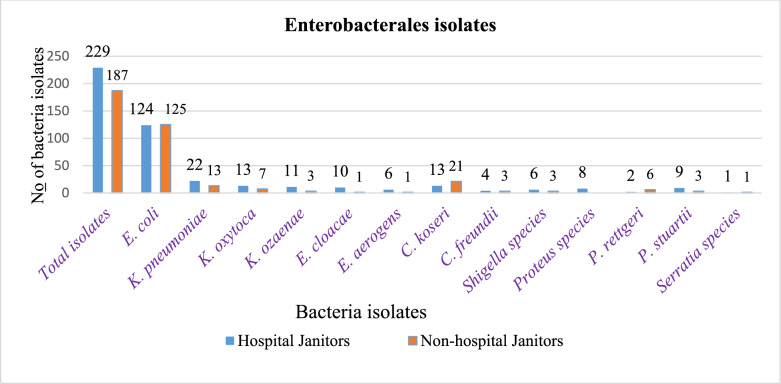
Fecal carriage rate of Enterobacterales isolate among hospital (N = 203) and non-hospital janitors (N = 182) at the UoG, Northwest Ethiopia, June to September 2025.

Among 385 janitors, a total of 416 isolates were recovered, with 229 from hospital janitors and 187 from non-hospital janitors. While single-organism cultures were the most frequent outcome in both groups, co-colonization with two organisms was over five times more common among hospital janitors (n = 26) than non-hospital janitors (n = 5). *Escherichia coli* was the predominant organism in both groups, constituting 124 (54.1%) of isolates from hospital janitors and 125 (66.8%) from non-hospital janitors. There was no statistically significant difference in the fecal carriage rate of *E. coli* isolate among hospital and non-hospital janitors (*p* = 0.119). The profile of secondary pathogens, however, differed. *Klebsiella pneumoniae* was the second most common isolate among hospital janitors 22 (9.6%). In contrast, among non-hospital janitors, *C. koseri* 21 (11.2%) was the second most common isolate (Fig. 2).

### Antimicrobial resistance pattern of Enterobacterales isolates from hospital and non-hospital janitors

The antimicrobial susceptibility of the isolates was tested against thirteen antibiotics from eight classes. High resistance rates were observed to tetracycline and gentamicin, with isolates from hospital janitors (64.2% and 58.1%, respectively) than those isolates from non-hospital janitors (48.1% and 34.8%, respectively). In contrast, several antibiotics remained highly effective. For isolates from hospital janitor, the most effective agents were amoxicillin-clavulanic acid (94.3%) and ciprofloxacin (90.8%). However, for isolates from non-hospital janitor, the most effective agents were meropenem (97.3%) and cefoxitin (94.1%) (Table 4).

**Table 4:** Antimicrobial resistance patterns of Enterobacterales isolates from hospital (N = 203) and non-hospital janitors (N = 182) at the UoG, Northwest Ethiopia, June to September 2025.

Class of antibiotics		Beta-lactams				Beta-lactam combination group	Carbapenems		Aminoglycosides		Tetracycline	Phenicols	Fluoroquinolones	Sulfonamides
Antibiotics		CAZ	CRO	CTX	CXT	AMC	MER	IMP	GEN	AK	TET	CHL	CIP	SXT
*E. coli* (N = 249)	HJ(124) R (n (%))	55(44.4)	52(41.9)	48(38.7)	24(19.4)	7(5.6)	10(8.1)	19(15.3)	67(54.0)	45(36.3)	77(62.1)	27(21.8)	10(8.1)	55(44.4)
	NHJ(125) R (n (%))	42(33.6)	30(24.0)	27(21.6)	7(5.6)	6(4.8)	0	12(9.6)	39(31.2)	12(9.6)	57(45.6)	18(14.4)	5(4.0)	32(25.6)
*K. pneumoniae* (N = 35)	HJ(22) R (n (%))	11(50.00)	10(45.45)	11(50)	8(36.4)	3(13.6)	9(40.9)	11(50)	16(72.7)	14(63.6)	19(86.4)	10(45.5)	7(31.8)	14(63.6)
	NHJ(13) R (n (%))	5(38.46)	5(38.46)	4(30.8)	1(7.7)	3(23.1)	2(15.4)	4(30.8)	5(38.5)	3(23.1)	7(53.8)	8(61.5)	4(30.8)	4(30.8)
*K. ozaenae* (N = 14)	HJ = 11 R (n (%))	5(45.45)	4(36.36)	4(36.36)	0	2(18.2)	2(18.2)	4(36.4)	7(63.6)	6(54.5)	8(72.7)	2(18.2)	0	8(72.7)
	NHJ = 3 R (n (%))	1(33.33)	1(33.33)	1(33.33)	0	0	1(33.3)	1(33.3)	2(66.7)	0	1(33.3)	2(66.7)	0	0
*K. oxytoca* (N = 20)	HJ = 13 R (n (%))	9(69.23)	5(38.46)	5(38.46)	1(7.7)	0	1(7.7)	2(15.4)	10(76.9)	6(46.2)	10(76.9)	3(23.1)	1(7.7)	8(61.5)
	NHJ = 7 R (n (%))	2(28.57)	0	0	0	0	1(14.3)	0	4(57.1)	0	6(85.7)	1(14.3)	0	2(28.6)
*E. cloacae* (N = 11)	HJ = 10 R (n (%))	4(40.0)	3(30.0)	4(40.0)	2(20.0)	1(10.0)	2(20.0)	4(40.0)	7(70.0)	8(80.0)	7(70.0)	3(30.0)	0	6(60.0)
	NHJ = 1 R (n (%))	0	0	0	0	0	0	0	1(100.0)	0	0	0	0	0
*E. aerogens* (N = 7)	HJ = 6 R (n (%))	4(66.67)	2(33.3)	1(16.7)	0	0	0	4(66.7)	3(50)	2(33.3)	6(100)	0	0	2(33.3)
	NHJ = 1 R (n (%))	0	1(100)	0	0	0	0	0	0	0	0	0	0	1(100)
*C. koseri* (N = 34)	HJ = 13 R (n (%))	2(15.38)	3(23.1)	1(7.7)	1(7.7)	0	0	1(7.7)	7(53.8)	5(38.5)	7(53.8)	1(7.7)	0	8(61.5)
	NHJ = 21 R (n (%))	3(14.29)	1(4.8)	3(14.3)	0	0	0	0	9(42.9)	1(4.8)	11(52.4)	1(4.8)	1(4.8)	5(23.8)
*C. freundii* (N = 7)	HJ = 4 R (n (%))	0	1(25.0)	0	0	0	0	0	1(25)	0	2(50.0)	1(25.0)	1(25.0)	1(25)
	NHJ = 3 R (n (%))	0	3(100.0)	2(66.7)	2(66.7)	1(33.3)	0	0	0	2(66.7)	1(33.3)	3(100)	0	1(33.3)
*Shigella* species (N = 9)	HJ = 6 R (n (%))	0	0	2(33.3)	1(16.7)	0	0	0	1(16.7)	1(16.7)	3(50.0)	0	2(33.3)	0
	NHJ = 3 R (n (%))	0	0	0	0	1(33.3)	0	0	0	0	0	0	0	0
*Proteus* species (N = 8)	HJ = 8 R (n (%))	0	0	0	0	0	0	1(12.5)	4(50)	1(12.5)	1(12.5)	1(12.5)	0	0
*P. stuartii* (N = 12)	HJ = 9 R (n (%))	2(22.2)	2(22.2)	1(11.1)	0	0	0	0	7(77.8)	1(11.1)	5(55.6)	0	0	3(33.3)
	NHJ = 3 R (n (%))	0	0	0	0	0	0	0	0	0	1(33.3)	1(33.3)	0	0
*P. rettgeri* (N = 8)	HJ = 2 R (n (%))	2(100.0)	1(50.0)	0	0	0	0	0	2(100.0)	0	2(11.0)	0	0	0
	NHJ = 6 R (n (%))	4(66.7)	4(66.7)	2(33.3)	1(16.7)	2(33.3)	1(16.7)	4(66.7)	4(66.7)	2(33.3)	5(83.3)	4(66.7)	2(33.3)	5(83.3)
*Serratia* species (N = 2)	HJ = 1 R (n (%))	0	0	0	0	0	0	0	1(100.0)	0	0	0	0	0
	NHJ = 1 R (n (%))	0	0	0	0	0	0	0	1(100.0)	1(100.0)	1(100.0)	0	0	1(100.0)
Total (N = 416)	HJ = 229 R (n (%))	**94(41.0)**	**83(36.2**	**77(30.1)**	**37(16.2)**	**13(5.7)**	**24(10.5)**	**46(20.1)**	**133(58.1)**	**89(38.9)**	**147(64.2)**	**45(20.4)**	**21(9.2)**	**105(45.9)**
	NHJ = 187 R (n (%))	**57(30.5)**	**45(24.1)**	**39(20.9)**	**11(5.9)**	**13(7.0)**	**5(2.7)**	**21(11.2)**	**65(34.8)**	**21(11.2)**	**90(48.1)**	**38(20.3)**	**12(6.4)**	**51(27.3)**

HJ = Hospital Janitors; NHJ = Non-hospital Janitors; R = Resistance.

*CXT* Cefoxitin, *CRO* Ceftriaxone, *CAZ* Ceftazidime, *CTX* cefotaxime, *AMC* Amoxicillin-clavulanic acid, *MER* Meropenem, *IMP* Imipenem, *GEN* Gentamycin, *AK* Amikacin, *TE* Tetracycline, *CHL* Chloramphenicol, *CIP* Ciprofloxacin, *SXT* Sulphamethoxazole-trimethoprim.

### Multidrug resistance profiles of Enterobacterales isolates among hospital and non-hospital Janitors

Multidrug resistance (MDR) was determined based on resistance to eight classes of antibiotics: beta-lactams, β-lactam combinations, carbapenems, aminoglycosides, tetracyclines, phenicols, fluoroquinolones, and sulfonamides. The overall fecal carriage rate of MDR Enterobacterales among janitors was 54% (95% CI: 48.9, 59.1). This rate was significantly higher in hospital janitors compared to non-hospital janitors ((63.5% (95% CI: 56.5, 70.2) vs. 43.4% (95% CI: 36.1, 50.9), respectively; *p* < 0.001)).

Multidrug-resistant Enterobacterales was found in 133 (58.1%) isolates from hospital janitors and 79 (42.2%) from non-hospital janitors. Additionally, a four hospital janitors were co-colonized by two MDR isolates. Furthermore, a few isolates exhibited resistance to all eight classes; these included one *E. coli* and one *K. pneumoniae* from hospital janitors, as well as one *K. pneumoniae* and one *P. rettgeri* from non-hospital janitors (Table 5).

**Table 5:** Multidrug resistance profiles of Enterobacterales isolates among hospital (N = 203) and non-hospital janitors (N = 182) at the UoG, Northwest Ethiopia, June to September 2025.

Enterobacterales isolates (N = 416)		Level of resistance (n)									
		R0	R1	R2	R3	R4	R5	R6	R7	R8	MDR ≥ R3
*E. coli* (N = 249)	HJ = 124	23	17	14	24	23	9	10	3	1	70 (56.5%)
	NHJ = 125	54	11	11	25	14	5	5	0	0	49 (39.2%)
*K. pneumoniae* (N = 35)	HJ = 22	1	1	2	3	4	6	2	2	1	18 (81.8%)
	NHJ = 13	1	2	1	2	2	3	1	0	1	9 (69.2%)
*K. oxytoca* (N = 20)	HJ = 13	2	1	0	4	3	1	2	0	0	10 (76.9%)
	NHJ = 7	1	0	2	4	0	0	0	0	0	4 (57.1%)
*K. ozaenae* (N = 14)	HJ = 11	2	1	1	0	4	1	1	1	0	7 (63.6%)
	NHJ = 3	1	0	1	0	1	0	0	0	0	1 (33.3%)
*E. cloacae* (N = 11)	HJ = 10	0	1	0	3	3	3	0	0	0	9 (90%)
	NHJ = 1	0	1	0	0	0	0	0	0	0	0
*E. aerogens* (N = 7)	HJ = 6	1	0	0	2	2	1	0	0	0	5 (83.3%)
	NHJ = 1	0	0	1	0	0	0	0	0	0	0
*C. koseri* (N = 34)	HJ = 13	2	2	3	4	2	0	0	0	0	6 (46.2%)
	NHJ = 21	6	6	3	5	0	1	0	0	0	6 (28.6%)
*C. freundii* (N = 7)	HJ = 4	1	0	2	1	0	0	0	0	0	1 (25.0%)
	NHJ = 3	0	0	0	2	1	0	0	0	0	3 (100.0%)
*Shigella* species (N = 9)	HJ = 6	2	1	1	2	0	0	0	0	0	2 (33.3%)
	NHJ = 3	2	1	0	0	0	0	0	0	0	0
*Proteus* species (N = 8)	HJ = 8	3	3	2	0	0	0	0	0	0	0
*P. rettgeri* (N = 8)	HJ = 2	0	0	0	2	0	0	0	0	0	2 (100.0%)
	NHJ = 6	0	0	0	1	1	1	2	0	1	6 (100.0%)
*P. stuartii* (N = 12)	HJ = 9	2	1	3	2	1	0	0	0	0	3 (33.3%)
	NHJ = 3	2	0	1	0	0	0	0	0	0	0
*Serratia* species (N = 2)	HJ = 1	0	1	0	0	0	0	0	0	0	0
	NHJ = 1	0	0	0	1	0	0	0	0	0	1 (100.0%)
Total (N = 416)	HJ = 229	39	29	28	47	42	21	15	6	2	**133(58.1%)**
	NHJ = 187	67	21	20	40	19	10	8	0	2	**79 (42.2%)**

HJ = Hospital Janitors; NHJ = Non-hospital Janitors.

R0 = sensitive for all eight classes of antibiotics; R1 = resistant for only one class of antibiotics; R2 = resistant for two classes of antibiotics; R3 = resistant for three classes of antibiotics, etc.; *MDR* multidrug-resistant.

### Distribution of MDR Enterobacterales isolates among eight working sites of hospital janitors at the UoGCSH

The prevalence of MDR isolates varied across the eight hospital sites studied. The highest rates were observed in the ICU (76.9%), followed by the laboratory (75.0%) and the emergency room (61.1%). Lower prevalence was found in the outpatient wards (60.0%), other departments (57.1%), the pediatric wards (53.8%), the inpatient wards (52.9%), and the operation room (52.4%) (Table 6).

**Table 6:** Distribution of MDR Enterobacterales isolates among eight working sites of hospital janitors at the UoGCSH, Northwest Ethiopia, June to September 2025 (N = 203).

Enterobacterales isolates (N = 229)	UoGCSH study sites								
	Outpatient wards (N = 45)	Inpatient wards (N = 34)	Emergency room (N = 18)	ICU (N = 13)	Laboratory room (N = 8)	Operation room (N = 42)	Pediatric wards (N = 13)	Other departments (N = 56)	Total (N = 229)
	MDR N (%)	MDR N (%)	MDR N (%)	MDR N (%)	MDR N (%)	MDR N (%)	MDR N (%)	MDR N (%)	MDR N (%)
*E. coli* (N = 124)	9 (7.3)	11 (8.9)	5 (4.0)	4 (3.2)	4 (3.2)	14 (11.3)	5 (4.0)	18 (14.5)	**70 (56.5)**
*K. pneumoniae* (N = 22)	3 (13.6)	1 (4.5)	3 (13.6)	4 (18.2)	NR	2 (9.1)	NR	5 (22.7)	**18 (81.8)**
*K. oxytoca* (N = 13)	3 (23.1)	4 (30.8)	NR	NR	1 (7.7)	1 (7.7)	0	1 (7.7)	**10 (76.9)**
*K. ozaenae* (N = 11)	0	1 (9.1)	1 (9.1)	1 (9.1)	NR	1 (9.1)	NR	3 (27.3)	**7 (63.6)**
*E. cloacae* (N = 10)	5 (50.0)	NR	NR	NR	1 (10.0)	2 (20.0)	1 (10.0)	NR	**9 (90.0)**
*E. aerogens* (N = 6)	3 (50.0)	1 (16.7)	NR	NR	NR	NR	NR	1 (16.7)	**5 (83.3)**
*C. koseri* (N = 13)	2 (15.4)	0	1 (7.7)	NR	NR	1 (7.7)	0	2 (15.4)	**6 (46.2)**
*C. freundii* (N = 4)	NR	0	NR	NR	NR	1 (25.0)	NR	0	**1 (25.5)**
*Shigella* species (N = 6)	1 (16.7)	0	NR	NR	NR	0	NR	1 (16.7)	**2 (33.3)**
*Proteus* species (N = 8)	0	NR	NR	NR	NR	0	0	0	**0**
*P. rettgeri* (N = 2)	NR	NR	NR	NR	NR	NR	1 (50.0)	1 (50.0)	**2 (100.0)**
*P. stuartii* (N = 9)	1 (11.1)	0	1 (11.1)	1 (11.1)	NR	0	0	0	**3 (33.3)**
*Serratia* species (N = 1)	NR	0	NR	NR	NR	NR	NR	NR	**0**
Total N (%)	**27 (60.0)**	**18 (52.9)**	**11 (61.1)**	**10 (76.9)**	**6 (75.0)**	**22 (52.4)**	**7 (53.8)**	**32 (57.1)**	**133 (58.1)**

*NR* not reported (No bacterial isolates available to determine MDR profile).

*UoGCSH* University of Gondar comprehensive specialized hospital; MDR = Multidrug-resistant, *ICU* Intensive care unit.

Other departments = including; Gynecology and Obstetrics Ward, Psychiatry department, Oncology ward, Leishmaniasis research and treatment center, etc.

### Distribution of MDR Enterobacterales isolates among non-hospital janitors across three UoG campuses

The prevalence of MDR isolates among non-hospital janitors showed little variation across the three UoG campuses included in the study. The highest rates were observed in the Maraki campus (43.6%), followed by the Atse Fasile campus (43.2%), and the Atse Tewodros campus (41.1%), (Table 7).

**Table 7:** Distribution of MDR Enterobacterales isolates among non-hospital janitors across three UoG campuses, Northwest Ethiopia, June to September 2025 (N = 182).

Enterobacterales isolates (N = 187)	UoG non-hospital study sites			
	Atse tewodros campus (N = 95)	Maraki campus (N = 55)	Atse Fasile campus (N = 37)	Total (N = 187)
	MDR N (%)	MDR N (%)	MDR N (%)	MDR N (%)
*E. coli* (N = 125)	24 (19.2)	14 (11.2)	11 (8.8)	**49 (39.2)**
*K. pneumoniae* (N = 13)	7 (53.8)	NR	2 (15.4)	**9 (69.2)**
*K. oxytoca* (N = 7)	3 (42.9)	NR	1 (14.3)	**4 (57.1)**
*K. ozaenae* (N = 3)	0	1 (33.3)	NR	**1 (33.3)**
*E. cloacae* (N = 1)	NR	NR	0	**0**
*E. aerogens* (N = 1)	0	NR	NR	**0**
*C. koseri* (N = 21)	0	6 (28.6)	0	**6 (28.6)**
*C. freundii* (N = 3)	3 (100.0)	NR	NR	**3 (100.0)**
*Shigella* species (N = 3)	0	0	0	**0**
*P. rettgeri* (N = 6)	2 (33.3)	3 (50.0)	1 (16.7)	**6 (100.0)**
*P. stuartii* (N = 3)	0	NR	NR	**0**
*Serratia* species (N = 1)	NR	NR	1 (100.0)	**1 (100.0)**
**Total N (%)**	**39 (41.1)**	**24 (43.6)**	**16 (43.2)**	**79 (42.2)**

*NR* not reported (No bacterial isolates available to determine MDR profile).

*UoG* University of Gondar, *MDR* Multidrug-resistant.

### Factors associated with fecal carriage of MDR Enterobacterales among Janitors

Variables with a *p*-value of ≤ 0.25 in the bivariable analysis were included in the multivariable logistic regression model. The final model, identified three factors significantly associated with MDR Enterobacterales carriage. Antibiotic use within the past three months (AOR: 2.35; 95% CI: 1.42, 3.90). Furthermore, a history of UTI in the past three months (AOR: 2.02; 95% CI: 1.12, 3.64), and a history of GIT symptoms in the past three months (AOR: 2.04; 95% CI: 1.15, 3.60) were also significantly associated with increased odds of MDR Enterobacterales carriage (Table 8).

**Table 8:** Factors associated with fecal carriage of MDR Enterobacterales among janitors at the UoG, Northwest Ethiopia, June to September 2025 (N = 385).

Variables	Category	MDR Enterobacterales N (%)		Bivariable analysis		Multivariable analysis	
		Positive (N = 208)	Negative (N = 177)	COR (95% CI)	*P*-value	AOR (95% CI)	*P*- value
UoG work area	Hospital	129 (63.5%)	74 (36.5%)	2.27 (1.51, 3.42)	< 0.001	1.36 (0.76, 2.43)	0.306
	Non-hospital	79 (43.4%)	103 (56.6%)	1		1	
Family size	1–5	196 (53.3%)	172 (46.7%)	1		1	
	> 5	12 (70.6%)	5 (29.4%)	2.11 (0.73, 6.1)	0.170	0.83 (0.25, 2.71)	0.756
Exposure to contaminated surfaces	Frequently	46 (49.5%)	47 (50.5%)	1.11 (0.64, 1.91)	0.719	0.83 (0.44, 1.56)	0.555
	Occasionally	108 (61.0)%	69 (39.0%)	1.77 (1.1, 2.84)	0.019	1.22 (0.4, 2.12)	0.492
	Never	54 (47.0%)	61 (53.0%)	1		1	
PPE	Yes	126 (53.4%)	110 (46.6%)	1		1	
	Sometimes	70 (52.2%)	64 (47.8%)	0.96 (0.62, 1.46)	0.831	1.15 (0.66, 2.01)	0.617
	No	12 (80.0%)	3 (20.0%)	3.50 (0.96, 12.70)	0.058	2.69 (0.66, 10.97)	0.168
Safe toilet access at home	Yes	177 (51.9%)	164 (48.1%)	1		1	
	No	31 (70.5%)	13 (29.5%)	2.21 (1.12, 4.37)	0.023	1.94 (0.93, 4.05)	0.079
Fingernail status	Trimmed	147 (51.4%)	139 (48.6%)	1		1	
	Not-trimmed	61 (61.6%)	38 (38.4%)	1.52 (0.95, 2.42)	0.080	0.83 (0.47, 1.44)	0.503
Antibiotics used without prescription in the last 3 month	Yes	71 (62.8%)	42 (37.2%)	1.67 (1.06, 2.61)	0.026	1.37 (0.82, 2.30)	0.231
	No	137 (50.4%)	136 (49.6%)	1		1	
Prescribed antibiotics used in the last 3 month	Yes	109 (72.7%)	41 (27.3%)	3.65 (2.35, 5.69)	< 0.001	**2.35 (1.42, 3.9)**	0.001*
	No	99 (42.1%)	136 (57.9%)	1		1	
History of GI symptom in the last 3 month	Yes	73 (73.0%)	27 (27.0%)	3.0 (1.82, 4.95)	< 0.001	**2.04 (1.15, 3.6)**	0.015*
	No	135 (47.4%)	150 (52.6%)	1		1	
History of UTI symptom in the last 3 month	Yes	60 (73.2%)	22 (26.8%)	2.86 (1.67, 4.89)	< 0.001	**2.02 (1.12, 3.64)**	0.020*
	No	148 (48.8%)	155 (51.2%)	1		1	
Close contact with hospitalized person in the last 3 month	Yes	101 (62.7%)	60 (37.3%)	1.84 (1.22, 2.78)	0.004	1.22 (0.76, 1.97)	0.406
	No	107 (47.8%)	177 (52.2%)	1		1	

**Bold** and * = Statistically significant at *p*-value < 0.05, 1 = Reference group.

*MDR* multidrug resistant, *COR* crude odds ratio, *AOR* adjusted odds ratio, *CI* confidence interval, *UTI* urinary tract infection, *GI* gastro intestinal, *UoG* University of Gondar, *PPE* personnel protective equipment.

## Discussion

Fecal carriage of MDR Enterobacterales isolates are a growing public health threat worldwide, contributing to both subsequent infections in carriers and potential person-to-person transmission^35,36^. Despite this, data on fecal carriage rates of MDR Enterobacterales among janitors in Ethiopia remain scarce.

In our study, *Escherichia coli* was the dominant isolate, 54.1% of isolates from hospital janitors and 66.8% of isolates from non-hospital janitors. This prevalence of *E. coli* agrees with study results reported from food handlers in Gondar, Ethiopia^22^, and community population in Addis Ababa, Ethiopia^37^. This finding highlighting its role as a common commensal and potential pathogen.

According to the present study, Enterobacterales isolates showed high rate of resistance to tetracycline and gentamicin among hospital (64.2 and 58.1%, respectively) compared to non-hospital janitors (48.1 and 34.8%, respectively). The higher resistance rate among hospital janitors is likely attributable to their greater occupational exposure to environmental contaminants within healthcare settings and increased selective pressure from antibiotics^38^. Our study also found that the most effective agents for isolates from hospital janitors were amoxicillin-clavulanic acid (94.3%) and ciprofloxacin (90.8%), which was comparable with the study of HCWs in Egypt^39^. However, the most effective agent for isolates from non-hospital janitors was meropenem (97.3%), which was comparable with the study results from food handlers in Gondar, Ethiopia^22^.

In our study, the overall fecal carriage rate of MDR Enterobacterales among janitors was 54% (95% CI 48.9, 59.1).This finding aligns with a study conducted in a slaughterhouse in Nigeria (50%)^40^ and among farmers in Uganda (49.4%)^41^. However, it is higher than rates reported among food handlers in Gondar, Ethiopia (42.3%)^22^ and Dilla, Ethiopia (16.8%)^42^, as well as in Kenya (30%)^43^, and a community-based study in Addis Ababa, Ethiopia (28.8%)^37^. This high MDR rate among janitors might be due to occupational exposure to high-risk sources of AMR. Janitors working in healthcare or high-density sanitation environments are frequently exposed to contaminated surfaces, biomedical waste, and fecal matter without consistent adherence to strict IPC. This exposure increases their risk of intestinal colonization by resistant bacteria^44^.

In the present study, the MDR Enterobacterales rate was significantly higher in hospital janitors compared to non-hospital janitors ((63.5% (95% CI 56.5, 70.2) vs. 43.4% (95% CI 36.1, 50.9), respectively; *p* < 0.001)). This high MDR rate among hospital janitors is likely due to hospital settings are recognized reservoirs of MDR bacteria due to high antibiotic selective pressure and contaminated surfaces^45^.

Multivariable analysis in our study revealed a significant risk factors for MDR Enterobacterales colonization. Among janitors, colonization was independently associated with antibiotic use within the past three months, a history of UTI, and GIT symptoms in the past three months.

A history of antibiotic use within the last three months was associated with a more than twofold increased odds of harboring MDR Enterobacterales (AOR: 2.35; 95% CI 1.42, 3.90). This can be due to antibiotic use exerts a powerful selective pressure that eliminates susceptible gut bacteria, allowing pre-existing or newly acquired MDR Enterobacterales to proliferate and colonize^46^. Moreover, a history UTI within the last three months was associated with a more than twofold increased odds of harboring MDR Enterobacterales (AOR: 2.02; 95% CI 1.12, 3.64). This is primarily driven by prior antibiotic therapy, which exerts selective pressure on gut microbiota and promotes resistant strain persistence. Recurrent healthcare contact during UTI management further increases acquisition risk^47^. Furthermore, a history GIT symptoms within the last three months was associated with twofold increased odds of harboring MDR Enterobacterales (AOR: 2.04; 95% CI 1.15, 3.60). Gastrointestinal symptoms frequently prompt antibiotic therapy, which reduces microbial diversity and impairs the gut’s functional integrity, thereby facilitating the acquisition and prolonged carriage of AMR Enterobacterales strains^48^. This dysbiosis specifically depletes protective commensals that normally suppress MDR Enterobacterales through nutrient competition and short-chain fatty acid production^49^.

These findings underscore the importance of integrating janitors into AMR surveillance and infection prevention strategies within a One Health framework.

### Limitations of the study

The present study has some limitations. First, its cross-sectional design prevents any inference of causality and did not allow for the follow-up treatment or monitoring of carriers. Second, risk factor data were collected via questionnaire and may be subject to recall bias. Third, the majority of participants were female, which may limit the generalizability of the findings to a broader population. Additionally, further characterization is needed to specifically identify extended spectrum beta-lactamase and carbapenemase producers. Finally, antibiotic resistance was determined phenotypically; confirmatory molecular detection of resistance genes was not possible due to limited resources.

## Conclusion

This study provides evidence that janitors in Ethiopia carry a significant burden of MDR Enterobacterales (54.0%), with hospital janitors (63.5%) exhibiting notably higher fecal carriage of MDR Enterobacterales than non-hospital janitors (43.4%). The key predictors of MDR Enterobacterales carriage were an antibiotic use in the past three months, history of UTI, and GIT symptoms in the past three months. These findings highlight janitors, particularly these working in hospital settings, as an important reservoir for MDR Enterobacterales carriage.

## Recommendations

### To the community (specifically for janitors and their families)

We advise the consistent practice of hand hygiene with soap and water, especially after work, before handling food, and after using the toilet. Additionally, use antibiotics responsibly, complete the full prescribed course and never share leftover antibiotics. Furthermore, participate in any training offered by their workplace or local health centers on infection prevention is also strongly encouraged.

### To the University of Gondar

It is essential to formally acknowledge that janitors in both hospital and campus settings face a significant occupational risk of carrying drug-resistant bacteria. We recommend the implementation of regular, practical IPC training tailored for janitors, covering proper hand hygiene, the use of PPE, safe waste handling. Furthermore, the university must ensure the reliable provision of necessary resources, including soap, water, hand sanitizer, and appropriate PPE at all work sites.

### For public health policy and practice

National and hospital AMR surveillance programs should consider explicitly including janitors as a high-risk occupational group for routine screening. Health bureaus and hospital administrations should design and implement IPC training for janitors, emphasizing proper hand hygiene.

### For future research

Subsequent studies should including Extended-spectrum β-lactamase, and carbapenemase-producing Enterobacterales, as well as employ whole-genome sequencing to identify the specific resistance genes and plasmids carried by janitors, tracing transmission routes by comparing them with clinical and environmental isolates. Prospective cohort studies are needed to establish causality between the identified risk factors and colonization. Furthermore, intervention studies assessing the impact of tailored hand hygiene and antibiotic stewardship programs on colonization rates in this group are crucial.

## Supplementary Information

Supplementary Information.

## Data Availability

All relevant data are within the manuscript and its supporting information files; for further information, please contact the corresponding author.

## Abbreviations

AMR: Antimicrobial resistance
AOR: Adjusted odds ratio
AST: Antimicrobial susceptibility test
ATCC: American type culture collection
CMHS: College of medicine and health sciences
COR: Crude odds ratio
GI: Gastrointestinal
GNB: Gram-negative bacteria
HAIs: Hospital-acquired infections
HCWs: Healthcare workers
ICU: Intensive care unit
IPC: Infection prevention and control
LMICs: Low and middle income countries
MHA: Mueller–Hinton agar
PI: Principal investigator
PPE: Personnel protective equipment
QC: Quality control
SBLS: School of biomedical and laboratory sciences
SPSS: Statistical package for social science
TSB: Tryptic soy broth
UK: United Kingdom
UoG: University of Gondar
UoGCSH: University of Gondar Comprehensive Specialized Hospital
UTI: Urinary tract infection
CDC: Centers for disease control and prevention
CI: Confidence interval
CLSI: Clinical and Laboratory Standards Institute
MDR: Multi-drug resistance/resistant
WHO: World Health Organization
